# A Comparative Analysis of the APRI, FIB4, and FibroScan Score in Evaluating the Severity of Chronic Liver Disease in Chronic Hepatitis B Patients in India

**DOI:** 10.7759/cureus.19342

**Published:** 2021-11-07

**Authors:** Sumit Rungta, Shweta Kumari, Kamlendra Verma, Ghulam Akhtar, Amar Deep, Suchit Swaroop

**Affiliations:** 1 Gastroenterology, King George's Medical University, Lucknow, IND; 2 Biochemistry, King George's Medical University, Lucknow, IND; 3 Medical Gastroenterology, King George's Medical University, Lucknow, IND; 4 Medical Gastroenterology, King George’s Medical University, Lucknow, IND; 5 Zoology, University of Lucknow, Lucknow, IND

**Keywords:** cirrhosis, fibrosis, fibrosis index based on four factors (fib-4), chronic hepatitis b (chb), aspartate transaminase-to-platelet ratio index (apri)

## Abstract

Background and aims

Non-invasive assessment methods to assess liver fibrosis are important tools where FibroScan or liver biopsy is not accessible. The aim of this study is to assess the efficacy and performance of the fibrosis index based on four factors (FIB-4) and aspartate transaminase-to-platelet ratio index (APRI) to evaluate liver fibrosis against FibroScan for the stages of liver fibrosis in patients of chronic liver disease due to chronic hepatitis B (CHB).

Methods

This was a cross-sectional study conducted in a tertiary care center in Uttar Pradesh, India, and the patients were enrolled between 2017 and 2020. During the study period, 520 patients with a confirmed diagnosis of chronic hepatitis B virus (HBV) infection were selected. Laboratory blood testing and FibroScan were performed in all patients with CHB. APRI and FIB-4 were calculated using a standard formula involving laboratory parameters.

Result

The performance of FIB-4 scores are nearly similar to APRI, with area under the curve (AUC) 0.753, (95% CI) (0.711-0.795) (p<0.0001) for ≥F2 fibrosis (significant fibrosis) and even better 0.851 (0.815-0.887) (p<0.0001) for the F4 fibrosis (cirrhosis) group. Both the tests are proven good to diagnose fibrosis but FIB-4 has more area under the receiver operating characteristic (AUROC) than APRI in each set, thus FIB-4 is considered better than APRI.

Conclusions

APRI and FIB-4 scores showed good performance in detecting patients without liver fibrosis as compared with FibroScan. Based on this study, FibroScan can be avoided in patients examined for the diagnosis of mild fibrosis and cirrhosis in the source constrained area.

## Introduction

Hepatitis B virus (HBV) is an infectious agent that infects the human liver cells and causes liver inflammation, which leads to chronic infection and severe problems such as liver cirrhosis (LC) or hepatocellular carcinoma (HCC) more frequently than the other types of hepatitis viruses. The World Health Organization estimated that the number of people exposed to the hepatitis B virus to be approximately 2 billion [1]; 240 million of whom are chronic carriers worldwide [2]. In 2015, globally, 887,000 deaths were approximated to be due to complications of chronic HB (CHB) virus infection [1,3]. HBV positivity in the Indian population ranges from 1.1% to 12.2% and based on some regional level studies, it is estimated that in India, about 40 million people are chronically infected with the hepatitis B virus [4]. Persistent viral replication leads to continuous necroinflammation and patients are at higher risk of cirrhosis, end-stage liver disease, hepatic decompensation, and hepatocellular carcinoma (HCC) [5].

Progressive hepatic fibrosis with the development of cirrhosis is a characteristic of almost all chronic liver diseases. In patients with CHB, the accurate stage of hepatic fibrosis is the most important predictor of disease progression and indicates the need for initiating antiviral therapy. For many years, liver biopsy has been considered the gold standard for the staging of fibrosis [6]. However, liver biopsy has several limitations, such as it is an invasive and painful procedure [7], with rare but potentially life-threatening complications [8], and prone to sampling errors [9-10]. Therefore, many patients with CHB are reluctant to undergo a liver biopsy so patients may not receive antiviral therapy at the right time. Therefore, these limitations have stimulated the search for non-invasive methods.

FibroScan has been introduced as a new, non-invasive method for the diagnosis of liver fibrosis. FibroScan is based on the principle of transient elastography (TE), that is, the propagation velocity of a wave through a homogenous tissue is proportional to its elasticity, which is correlated with the amount of fibrosis in the liver [7]. FibroScan is the most reliable non-invasive tool for the assessment of liver fibrosis but because of its high cost and non-availability in small cities, the use of this tool is limited mainly at tertiary health care facilities [11].

Many studies report that FibroScan could predict liver fibrosis accurately in patients with chronic hepatitis C (CHC) [12-13]. In recent years, few research studies have performed FibroScan for the evaluation of fibrosis in patients infected with CHB also [14-15]. However, these studies were mainly performed in European countries and the United States, and the results cannot be generalized to Indian patients with CHB.

Various methods including serum markers, such as AST platelet ratio index (APRI), FIB-4, and TE (FibroScan) [11,16], have been proposed for the non-invasive evaluation of fibrosis in CHB patients. APRI and FIB-4 scores are two other non-invasive methods that can diagnose advanced fibrosis and cirrhosis with high accuracy in chronic hepatitis B patients compared to liver biopsy [17].

There are many advantages of non-invasive tests for detecting fibrosis, such as easy-to-perform and widespread availability, almost free from complications, can be done in the outpatient setting, does not require specialized training, homogeneity because of automated measurements of their component variables, tools for automated computation of score available (phone apps), and easy to repeat at frequent intervals. Because of the risks, contraindications, and observer dependence associated with liver biopsy, non-invasive methods should be better to evaluate liver fibrosis. Few non-invasive methods, such as the fibrosis index based on four factors (FIB-4), sonographic transient elastography (FibroScan), and aspartate aminotransferase (AST), have been intensively developed and have improved evaluation of the LF (liver fibrosis) stages.

The aim of this study was to evaluate the effectiveness of FIB-4 and APRI to differentiate the stages of liver fibrosis against FibroScan-based staging of liver stiffness in patients with chronic hepatitis B (CHB) infection. We also tried to evaluate whether any non-invasive methods are superior among both of them or not.

## Materials and methods

Study period, population, and design

We performed a cross-sectional study from 01 January 2017 to 30 January 2020 in the department of medical gastroenterology, King George Medical University, Lucknow, Uttar Pradesh, India. During the study period, 520 patients with a confirmed diagnosis of chronic HBV infection were enrolled. This study was conducted following recommendations of the ethics research committee (registration no. ECR/262/Inst/UP/2013/RR-16) and ethical clearance (ethical approval ref. no. is 95th ECM II A/P27) was obtained from our institution. Written consent was also obtained from all the participants for conducting the present study.

All patients were asked about their exposure to risk factors (i.e. drug addiction, blood transfusion, major or minor surgeries, disease severity, and complications such as ascites, hepatic encephalopathy), presence of ascites, jaundice, hematemesis, melena, pedal edema, easy bruisability, bleeding gums, or recent use of any alternative medicine or alcohol. This study included both acute HBV and chronic HBV-infected patients with and without cirrhosis, as well as patients who were on antiviral therapy and follow-up patients who were not on antiviral therapy.

Patient’s history, physical examination, hematological and biochemical investigations like hemogram, liver function tests, serum protein and albumin tests, ultrasonography (USG), and TE were done in all the patients. Patients with the presence of other causes of liver disease, HCC, prior interferon therapy, human immunodeficiency virus (HIV), and co-infected with HBV, and liver transplantation were excluded from the study.

Laboratory methods

Hematological and biochemical parameters were determined using commercially available assays. All patients' samples were tested for HBsAg by using a commercial enzyme-linked immunoassay (ELISA) kit (Abbott Laboratories, Chicago, IL). All HBV-positive patients were further investigated for quantitative HBV DNA by reverse transcription-polymerase chain reaction (RT-PCR) (Applied Biosystems, CA). Further, the included patients were investigated for HBeAg and HBeAb.

Liver stiffness measurements

Transient elastography (FibroScan) was performed on all of the patients in this study (Equipment: FibroScan, 402 with Vibration Regulated Transient Elastography (VCTE) technology). According to the manufacturer's instructions, a test must have 10 correct FibroScan readings to be considered accurate.

Stages of liver fibrosis were classified as follows based on FibroScan scores: F0-F1 (7 kPa), F2 (7-8.99 kPa), F3 (9-12.49 kPa), and F4 or cirrhosis (12.50 kPa). For each patient, FIB-4 and APRI scores were calculated based on laboratory results and the values were rounded to two decimal places. To predict patients with fibrosis (METAVIR≥F2), a lower and upper cutoff value of 1.45 and 3.25 for FIB-4 and 0.5 and 1.5, respectively, for the APRI were used based on the available data from the WHO guideline. However, APRI values of 1.0 and 2.0 are the lower and upper cutoff for detecting patients with cirrhosis, respectively, while no cutoff value for FIB-4 has been found to be satisfactory for detecting cirrhosis (METAVIR F4) [18].

Non-invasive scores (APRI and FIB-4) were calculated using laboratory data based on the following formulas:

APRI = [{AST (IU/L) / Upper normal limit of AST (IU/L)}/Platelet count (109/L)]*100 [19]

FIB-4 score = Age (years) X AST (IU/L) / Platelet count (109/L) * ALT (IU/ L)1/2 [20]

Statistical analysis

IBM’s SPSS version 24.0 software was utilized to conduct statistical analysis and to draft the data. For descriptive analysis, median and interquartile range (IQR) were obtained, non-parametric continuous variables, and percentages and numbers were obtained for categorical variables. The diagnostic performance of APRI and FIB-4 scores was measured by the area under the receiver operating characteristic (ROC) curve. The balance between sensitivity (Se), specificity (Sp) for a particular value of the test to rule out, or rule in the patients of interest was obtained from the coordinates of the curve. Positive predictive values (PPV) and negative predictive values (NPV) were also obtained for the cutoff value of the test. Statistical significance was defined as p<0.01.

## Results

Baseline data

Out of the total 520 patients enrolled in this study, around two-thirds (73.8%) of patients were male and one-third (26.2%) were female; however, this difference is statistically insignificant (p=0.82). The mean age of the study population was 36.36±14.39 years. The baseline biochemical characteristics of the study population are presented in Table 1. The study population was divided into four groups according to fibrosis stage, and we found that nearly half the study population was with normal liver FibroScan values (F0-F1), one-fourth of the patients exhibited cirrhosis (F4), while the remaining one-fourth of the population had intermediate fibrosis (F2 and F3). The median score of FibroScan, APRI, and FIB-4 was 7.20, 0.67, and 1.36, respectively.

**Table 1:** Baseline characteristics of the study population AST: aspartate aminotransferase; ALT: alanine aminotransferase; APRI: aspartate transaminase-to-platelet ratio index; FIB-4: fibrosis-4

Characteristics	Total Patients (n = 520)
Females	136 (26.2%)
Males	384 (73.8%)
Age (Mean ± SD) in years	36.36±14.39
BMI (kg/m^2^)	21.16±3.78
Haemoglobin (gm/dl)	12.58±2.25
Bilirubin (mg/dL)	1.22±0.84
AST (IU/L)	59.22±45.71
ALT (IU/L)	63.96±53.89
ALP (IU/L)	256.07±119.02
Platelets (/mm^3^)	156946.73±65526.20
Albumin (gm/dl)	3.91±0.79
FibroScan Score (kPa)	12.76±13.80
Stages of liver fibrosis as per FibroScan, n (%)	
F0-F1 (<7 kPa)	248 (47.7%)
F2 (7-8.99 kPa)	100 (19.2%)
F3 (9-12.49 kPa)	42 (8.1%)
F4 (≥12.5 kPa)	130 (25.0%)
APRI score (Median)	0.67 (0.13-31.43)
FIB-4 score (Median)	1.36 (0.13-35.30)
FibroScan (Median)	7.20 (2.30-75.00)

For the study purposes, we made two sets of population groups. In the first set, we divided the study population into patients with normal liver (i.e. FibroScan value <7.00 kPa) and patients with any kind of liver fibrosis or cirrhosis (i.e fibroscan ≥7 kPa); the second set of non-cirrhotic patients (i.e. FibroScan value <12.5 kPa) were included in group 1 and cirrhotic patients included in group 2 (i.e. FibroScan value ≥12.5 kPa).

In set one, out of 520 patients, 248 (47.7%) had normal liver and 264 (52.3%) patients had fibrosis or cirrhosis while in set two, out of 520 patients, 390 (75%) patients were having non-cirrhotic liver and 130 (25%) had cirrhotic liver.

Diagnostic performance of APRI and FIB-4 for significant fibrosis and cirrhosis

**Figure 1:**
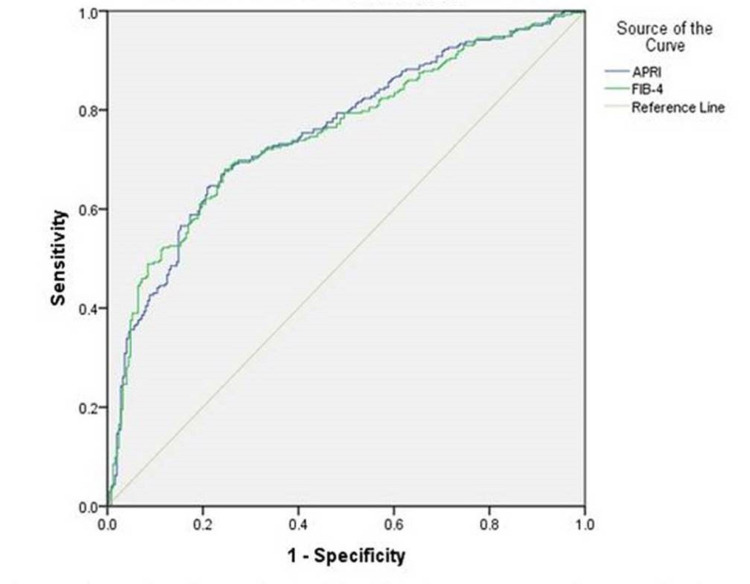
AUROC for APRI and FIB-4 for significant fibrosis AUC: area under the curve, APRI: aspartate aminotransferase-to-platelet ratio Index, FIB-4: fibrosis index based on four factors

**Figure 2:**
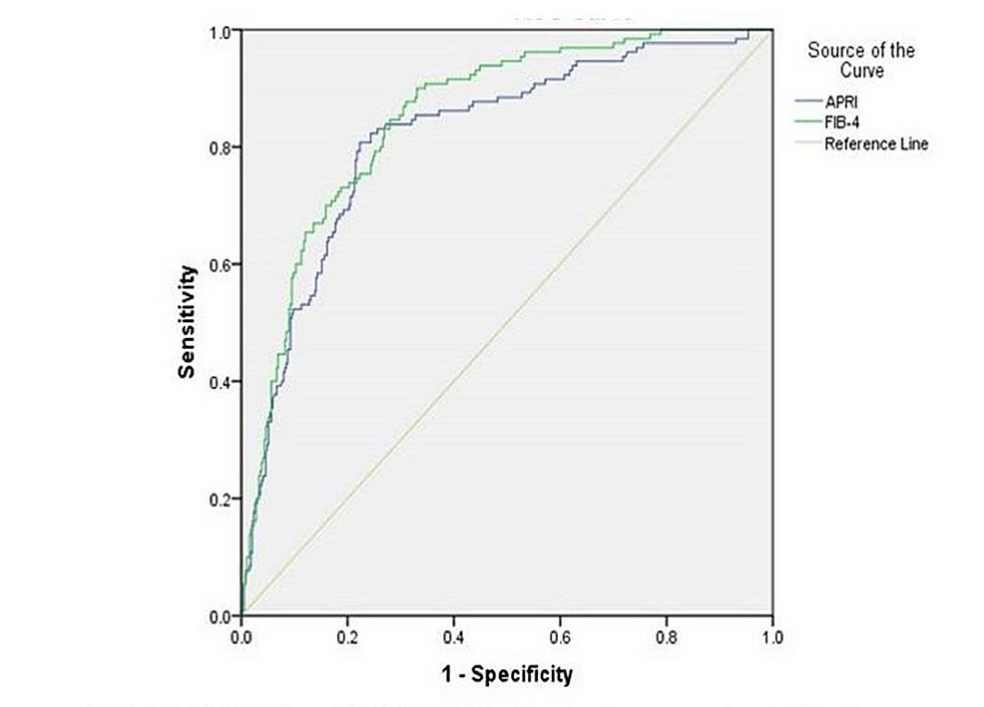
AUROC for APRI and FIB-4 for cirrhosis AUROC: area under the curve operating characteristic; APRI: aspartate aminotransferase-to-platelet ratio Index, FIB-4: fibrosis index based on four factors

High AUROC values for APRI and FIB-4 indicated the very good performance of these tests in recognizing significant fibrosis and cirrhosis. AUROC of APRI and FIB-4 to discover significant fibrosis (≥F2) were 0.756 (95% confidence interval [CI] 0.714-0.797) and 0.753 (95% CI 0.711-0.795), respectively. For the diagnosis of cirrhosis AUROC of APRI and FIB-4 were 0.818 (95% CI 0.776-0.861) and 0.815 (95% CI 0.815-0.887), respectively. The performance of APRI and FIB-4 on ROC are plotted in Table 2 and Figures 1-2.

**Table 2:** AUC and 95% CI of APRI and FIB-4 for liver fibrosis according to FibroScan APRI: aspartate aminotransferase-to-platelet ratio Index, FIB-4: fibrosis index based on four factors

Variable	≥F2 (Mild Fibrosis) (95% CI)	F4 (Cirrhosis) (95% CI)	p-value
APRI	0.756 (0.714-0.797)	0.818 (0.776-0.861)	<0.0001 (for all set)
FIB-4	0.753 (0.711-0.795)	0.851 (0.815-0.887)	<0.0001 (for all set)

The FIB-4 score are nearly similar to APRI, with AUC mean (95% CI) 0.753 (0.711-0.795) (p<0.0001) for significant fibrosis and even better, i.e. 0.851 (0.815-0.887) (p<0.0001) for the cirrhosis group (Table 2, Figures 1-2). Both the tests proved good to diagnose fibrosis but FIB-4 had more AUC than APRI in each set, thus FIB-4 showed better performance than APRI.

Sensitivity and specificity at upper and lower cut-offs for significant fibrosis and cirrhosis for both APRI and FIB-4 were calculated for the study population and compared with values proposed by the WHO HBV guidelines for the same (Tables 3-4). We also calculated PPV and NPV at the same cut-offs of APRI and FIB-4 for the significant fibrosis and cirrhosis groups. Since there is no defined value of upper and lower cutoff for FIB-4 to detect cirrhosis, therefore we tried to set the lower cutoff 1.75 and upper cut-off 4.00 to detect cirrhosis from the coordinate of AUC with sensitivity and specificity for lower cut-off are 80.0% and 73.8%, respectively, while sensitivity and specificity for the upper cut-off are 53.1% and 90.8%, respectively (Table 4).

**Table 3:** Performance of APRI and FIB-4 against METAVIR score for different cut-off values proposed by the WHO HBV guidelines WHO HBV guidelines [21] APRI: aspartate aminotransferase-to-platelet ratio Index, FIB-4: fibrosis index based on four factors; METAVIR: Meta-analysis of Histological Data in Viral Hepatitis; HBV: hepatitis B virus

Cut off values suggested by most of the Authors		≥F2		Cirrhosis (F4)	
		Sensitivity (95% CI)	Specificity (95% CI)	Sensitivity (95% CI)	Specificity (95% CI)
APRI	Lower cut-off (0.5 for ≥F2 and 1 for F4)	82% (77-86)	57% (49-65)	77% (73-81)	78% (74-81)
	Higher cut-off (1.5 for ≥F2 and 2 for F4)	39% (32-47)	92% (89-94)	48% (41-56)	94%(91-95)
FIB-4	Lower cut-off (1.45 for ≥F2)	89% (79-95)	42% (25-61)	-	-
	Higher cut-off (3.25 ≥F2)	59% (43-73)	74% (56-87)	-	-

**Table 4:** Upper and lower cut-offs of APRI and FIB-4 to detect fibrosis against FibroScan for each set of subgroups in the study population APRI: aspartate aminotransferase-to-platelet ratio index; FIB-4: fibrosis index based on four factors; PPV: positive predictive value; NPV: negative predictive value; * single best optimum value (≥F3)

	APRI			
	(Significant Fibrosis)		Cirrhosis (F4)	
Cut-off	0.5	1.5	1.0	2.0
Sensitivity	87.2%	54.7%	80.8%	51.5%
Specificity	48.9%	85.9%	76.9%	90.5%
PPV	45.7%	65.7%	53.8%	64.4%
NPV	88.5%	79.3%	92.3%	84.9%
	FIB-4			
	(Significant Fibrosis)		Cirrhosis (F4)	
Cut-off	1.45	3.25	1.75	4.0
Sensitivity	80.0%	56.4%	80.0%	53.1%
Specificity	69.8%	89.1%	73.8%	90.8%
PPV	57.3%	71.9%	50.5%	65.7%
NPV	88.7%	80.5%	91.7%	85.3%

## Discussion

Chronic liver disease (CLD) is a major public health issue that causes substantial morbidity and mortality. The progression and stage of liver fibrosis determine the prognosis and treatment of the disease. For clinical decision-making and follow-up, accurate quantification of liver fibrosis is of paramount importance. Only a liver histological examination can accurately confirm the existence of concomitant liver fibrosis, necroinflammatory activity, and steatosis. Liver biopsy is the gold standard test for evaluating liver fibrosis stages and due to the invasive nature of the procedure, the small size of the specimen, sampling risks, and inter-observer variability in the histopathological examination can restrict the routine use of liver biopsy [15]. Due to the non-invasive nature of transient elastography, several recommendations also state that it is an excellent way to assess liver fibrosis [2].

Due to the non-availability and high cost of transient elastography (FibroScan) in peripheral health centers made clinicians think of some other non-invasive reliable methods to determine liver fibrosis. APRI and FIB-4 are well-studied, non-invasive methods that can access liver fibrosis and cirrhosis [22].

In present study APRI identified significant fibrosis (p<0.0001) with a related AUC mean (95% CI) 0.756 (0.714-0.797) (Table 2, Figure 1), and cirrhosis (p<0.0001) with higher AUC mean 0.818 (0.776-0.861) (Table 2). Previously, some small-scale studies suggest that APRI and FIB-4 scores are higher in CHB patients with significant fibrosis (METAVIR staging) [23-24], which was also observed in this study. The advantage of this study includes a comparison with serum fibrosis models and using FibroScan as a reference.

We found the area under the receiver operating characteristic curve of APRI for significant fibrosis was 0.756. Our results are similar to a meta-analysis of 17 studies (n=3,573) that assessed APRI and found the area under the summary receiver operating characteristic (SROC) curve to be 0.77. In the same study, a summary receiver of operating characteristic curve of meta-analysis of 11 studies (N = 2,083) that assessed APRI for cirrhosis and found the area under the SROC curve to be 0.75 while our study finds AUROC for the same was 0.818, which is far better [22]. We also found the AUROC curve of FIB-4 for significant fibrosis and cirrhosis was 0.753 and 0.851, respectively, almost similar to findings of a meta-analysis of 10 studies (n = 1,996) that assessed FIB-4, and found the area under SROC curve to be 0.75 for significant fibrosis and SROC curve of meta-analysis of six studies (N = 1,304) that assessed FIB-4 for the cirrhosis, and found the area under the SROC curve to be 0.87 [22]. Our findings for APRI and FIB-4 in this study are also similar to Liu et al. and Mada PK et al. [25-26].

In this study, the ROC of FIB-4 was higher than that of APRI (0.851 vs 0.818; P=<0.0001) for diagnosing cirrhosis, which indicated FIB-4 might be more reliable than APRI as an indicator of cirrhosis in HBsAg-positive CHB patients with ALT≤5 ULN. Thus this study confirms the better performance of FIB-4 to predict cirrhosis with an AUROC of 0.851 in HBsAg-positive CHB patients.

For the detection of significant fibrosis, as described earlier, AUROC was similar for APRI and FIB-4 (0.756 vs 0.753, respectively) in this study but only sensitivity for lower and upper cut-offs of APRI are found to be better as compared to WHO for the same (87 vs 82% and 54.7 vs 39%) [21].

We also compared our study population between cirrhotic and non-cirrhotic at a lower cut-off of 1.0 and an upper cut-off of 2.0 for APRI as described by the WHO guideline against the METAVIR scoring system, and we found that sensitivity for lower cut-off (80.8 vs 77%), i.e. higher than WHO results, specificity for lower cut-off (76.9 vs 78%) is almost similar, while sensitivity and specificity for upper cut-off (51.5 vs 48% and 90.5 vs 94), i.e. almost near to the WHO guideline (Table 4 and Table 5). There is no exact cut-off to detect cirrhosis for FIB-4 proposed by WHO guidelines [21]. We calculated the lower cut-off, i.e. 1.75, and the upper cut-off, i.e. 4, to detect cirrhosis by FIB-4 from coordinates of the ROC curve with 80% sensitivity, 91.7% NPV for lower cut-off, and 90.8% specificity, 60.5% PPV for upper cut-off.

Some studies show different pathogenesis and different patterns of fibrosis according to different causes of chronic liver diseases and also justify the need for different cut-points of systems for the assessment of fibrosis from different causes [27-29].

APRI and FIB-4 were used primarily in resource-limited areas to predict liver fibrosis and cirrhosis [30]. In the clinical setting, the cut-offs with high specificity (i.e., fewer false-positive results) could be used to diagnose patients with significant fibrosis and cirrhosis, and the cut-offs with high sensitivity (i.e., fewer false-negative results) could be used to rule out the presence of significant fibrosis and cirrhosis.

Based on evidence from the systematic review, the WHO guidelines recommended that FibroScan and APRI were the most useful tests for the assessment of cirrhosis in resource-limited settings [21]. Our results suggest that APRI and FIB-4 are significantly able to overcome the limitations of Fibroscan in resource-limited areas. Based on our results, we recommended that FIB-4 should be considered as the preferred noninvasive fibrosis test, and FibroScan should be considered when FIB-4 is unavailable. Liver biopsy remains within the assemblage of hepatologists when there are discordances between clinical symptoms and the degree of fibrosis assessed by non-invasive approaches.

There may be a bias in the retrospective design of this study that might have caused selective bias resulting in underestimated sensitivity and overestimated specificity of APRI and FIB-4. Therefore, a larger sample with multicenter studies will be necessary to validate the new cut-offs of APRI and FIB-4.

## Conclusions

Since liver cirrhosis is the driving factor in CHB infection to determine the treatment regimen, duration, and follow-up strategy, the fibrosis test should be able to differentiate the maximum number of cirrhotic and mild fibrosis from normal or early stages of fibrosis. APRI and FIB-4 scores also showed good performance in detecting the patients without liver fibrosis compared with FibroScan. In conclusion, FIB-4 should be considered as the preferred non-invasive fibrosis test, and FibroScan should be considered when FIB-4 is unavailable. Based on this study, a liver biopsy could be avoided in patients examined for the diagnosis of significant fibrosis and cirrhosis.
